# Multi-Technique Diagnostic Analysis of Plasters and Mortars from the *Church of the Annunciation* (Tortorici, Sicily)

**DOI:** 10.3390/ma15030958

**Published:** 2022-01-26

**Authors:** Sebastiano Ettore Spoto, Giuseppe Paladini, Francesco Caridi, Vincenza Crupi, Sebastiano D’Amico, Domenico Majolino, Valentina Venuti

**Affiliations:** 1 Department of Mathematical and Computer Sciences, Physical Sciences and Earth Sciences, University of Messina, Viale Ferdinando Stagno D’Alcontres 31, I-98166 Messina, Italy; sebastiano.spoto@unime.it (S.E.S.); dmajolino@unime.it (D.M.); vvenuti@unime.it (V.V.); 2 Department of Chemical, Biological, Pharmaceutical and Environmental Sciences, University of Messina, Viale Ferdinando Stagno D’Alcontres 31, I-98166 Messina, Italy; vcrupi@unime.it; 3 Department of Geosciences, University of Malta, MSD2080 Msida, Malta; sebastiano.damico@um.edu.mt

**Keywords:** portable X-ray fluorescence spectrometry, portable Raman spectroscopy, ultramarine blue, mortars analysis, plasters analysis

## Abstract

Plasters and mortars of the *Church of the Annunciation* (Tortorici, Sicily) were characterized, for the first time, both at the elemental and molecular levels, by means of portable X-ray fluorescence (XRF) and Raman spectroscopy, to achieve information on the “state of health” of the whole structure. The understanding of their degradation mechanisms and the identification of consequent degradation patterns can define the environmental factors responsible for interpreting the potential pathological forms that can impact the general building vulnerability. In this sense, the results obtained in this article provide relevant information to identify and address both the characterization of building materials and the fundamental causes of their deterioration. At the same time, if coupled with the attempt to supply a chronological order of the major restoration interventions carried out on the investigated site, they provide new insights to calibrate the models for building vulnerability studies.

## 1. Introduction

The importance of multi-technique characterization of materials [1] is linked to the possibility to evaluate, on one side, any change in their physical state starting from the production of the specimen from the raw geological/mineralogical materials, and, on the other side, their diffusion through trade, use, and deployment in the archaeological record.

In the variety of techniques employed in different research areas of archaeometry [2,3,4,5], X-ray fluorescence (XRF) spectroscopy has an extensive use, being a well-established analytical method both in the laboratory and industry for the evaluation of the elemental composition. The employment of the XRF technique for the analysis of archaeological specimens lies in its remarkable combination of practical and economic advantages [6]. Many alternative techniques require dissolution procedures that are both time-consuming and costly in terms of the acids or other reagents required [6].

Raman spectroscopy is another analytical technique widely used in archaeometry [7,8], offering the advantage that, being a molecular spectroscopic technique, it can deal with inorganic and organic materials [3], including ancient objects and modern artists’ specimens [7].

For both methodologies, the development of mobile instrumentation is becoming available to the community to analyze art objects directly in situ [7,9,10,11,12].

A new and relatively modern approach in archaeometry consists of evaluating the seismic vulnerability of buildings. Ever-increasing attention towards cultural heritage safeguarding, especially in seismic areas, has been paid in order to avoid damage and/or collapse of historical buildings, with consequent loss of priceless heritage and human lives [13,14]. In this sense, evaluating potential local mechanisms is one of the most commonly used approaches for studying masonry buildings’ vulnerability [15]. The development of risk assessment procedures and management plans aimed at conserving cultural heritage involves experts with different backgrounds. In particular, in Italy, a multi-level approach consisting of three levels of evaluation has been defined, in order to assess seismic vulnerability of churches with an increasing refinement level [14].

The main body of this article comprises, therefore, the use of portable XRF and Raman spectroscopy to define the nature of the materials used during the realization of the *Church of the Annunciation* (hereinafter referred to as “Church”) at Tortorici. The obtained results represent a first step for the definition of the best pathway for the vulnerability assessment and usability of the investigated site, and provide, at the same time, useful information for planning future restoration strategies to be applied.

## 2. Materials and Methods

### 2.1. Site Description

The *Church of the Annunciation* is a masonry Roman Catholic Church located in the center of Tortorici, a municipality in the Metropolitan City of Messina (Sicily, Southern Italy). The Church, characterized by the specularity of the two façades as a unique and distinctive feature, is also known as “Batia” because the Abbey of the Poor Clares, a contemplative Order of nuns in the Catholic Church, was annexed to it.

The investigated Church belongs to a period of architectural transition in Sicily, between the XVIII and XIX centuries, where Baroque and neoclassical motifs were combined in the main Sicilian cities. Many of the religious buildings built in this period were destroyed or not completed, due to earthquakes, limited financial resources, and changes in taste by developers or designers. In the light of this, the investigated Church can be considered as a structure of significant level in the context of this peculiar rural and local architecture, up to now not well known or studied.

It is worth noting that only incomplete and inaccurate information regarding the Church’s role in local society are available. What is certainly known is that the Church served religious functions until 1963, when it was closed to the public due to its highly deteriorated state. Since then, the Church fell into a state of disrepair. Only at the end of the 20th century, the local authorities undertook a significant restructuring. Actually, the Church shows several extended fractures, of dubious origin, on its internal walls and no longer plays a role in the religious community, having become the center of artistic and cultural events in Tortorici.

**Figure 1:**
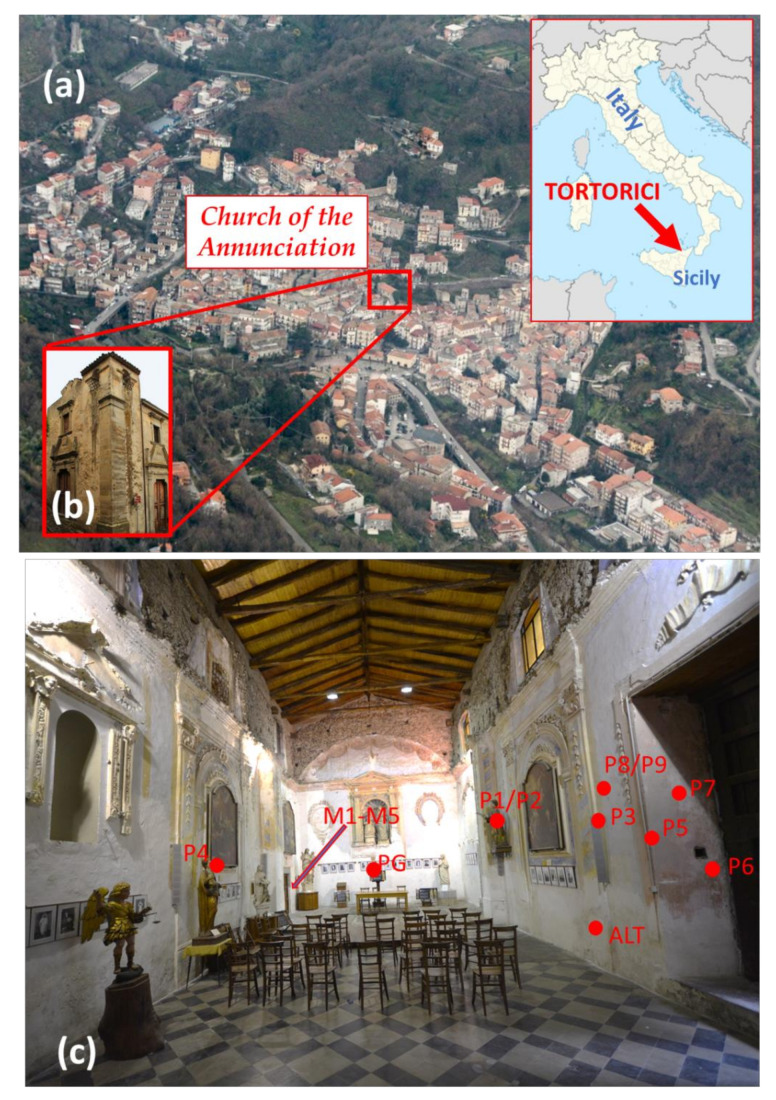
Geolocation map of the city of Tortorici, with the investigated Church indicated (**a**). The principal façades of the Church of the annunciation (**b**). Measurement areas of plasters and mortars at the Church. The measurement areas related to the mortars were taken around the indicated door (internal and external areas) that connects the Church with the former Poor Clare monastery (**c**).

A geolocation map of the city of Tortorici, with the investigated Church, are reported in Figure 1a,b, respectively.

### 2.2. Materials

A total of 16 points, representative of different pigmented plasters, preparatory ground, crystalline deposit of salts (efflorescence), and mortars of the interior of the Church, were investigated.

Table 1 reports a list of the analyzed points, together with their macroscopic description.

The exact positions of the investigated points within the Church are indicated in Figure 1c.

### 2.3. Methods

The investigation was performed in situ by using portable XRF and Raman instrumentations.

XRF measurements were carried out using a portable XRF Alpha 4000 analyzer (Innov-X systems, Inc., Woburn, MA, USA), which allows the detection of chemical elements with an atomic number (Z) between phosphorus and lead. It is equipped with a Ta anode X-ray tube as source and a Si PIN diode (active area of 170 mm^2^) as detector. For each point, two sequential tests were performed, the first with operating conditions of 40 kV and 7 µA and the second with 15 kV and 5 µA, for a total spectrum collection time of 120 s. The instrument was controlled by a Hewlett-Packard iPAQ Pocket PC (HP, Inc., Palo Alto, CA, USA), which was also used for the data storage. The calibration was performed using a soil light element analysis program (LEAP) II and was verified using alloy certified reference materials produced by Analytical Reference Materials International. When Cu is present, the L_α_ line of Ta is superimposed to the K_α_ line of Cu.

Raman measurements were performed by using a portable “BTR 111 Mini-RamTM” (B&W TEK Inc., Newark, NJ, USA) spectrometer with an excitation wavelength of 785 nm (diode laser), a maximum laser power of 280 mW at the excitation port, and a charge coupled device (CCD) detector (thermoelectric cooled, TE). The laser output power was continuously adjusted by maximizing the signal-to-noise ratio while minimizing the integration time. The laser spot size was 85 μm at a working distance of 5.90 mm. The maximum power at the samples was about 55 mW. All the spectra were registered in the wavenumber range of 60–3150 cm^−1^ by using an acquisition time of 320 s and a resolution of 8 cm^−1^ by accumulating several scans for each spectrum in order to improve the signal-to-noise ratio.

The abbreviations of minerals reported in the present study follow the list approved by the International Mineralogical Association (IMA) Commission on New Minerals, Nomenclature and Classification (CNMNC) [16].

## 3. Results and Discussion

### 3.1. Plasters

**Figure 2:**
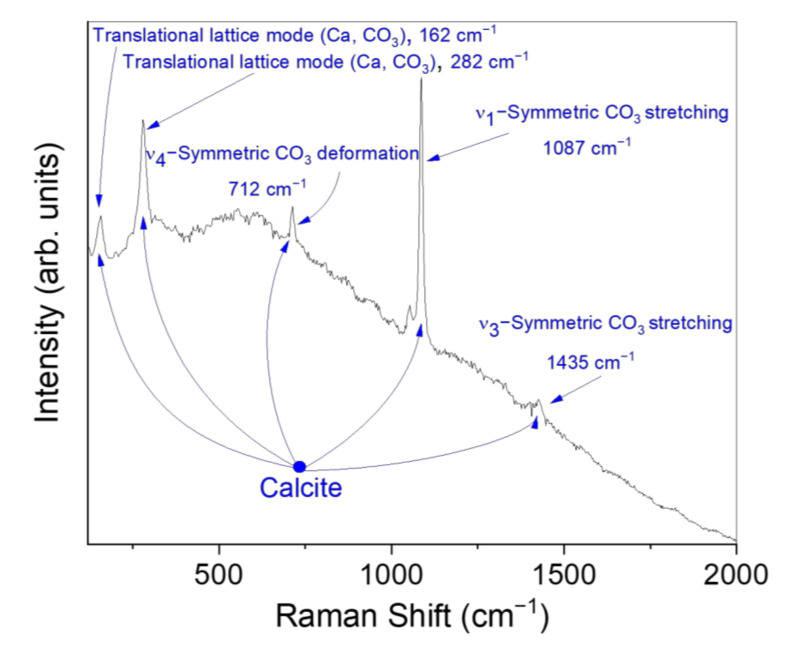
Raman spectrum recorded on the preparatory ground.

As far as the preparation ground is concerned, identified as point PG, the Raman spectrum collected on it (Figure 2) revealed an intense peak at ~1080 cm^−1^, along with other contributions at ~162, ~282, ~712, and ~1435 cm^−1^, all associated to the presence of calcite, the most stable polymorph of calcium carbonate (CaCO_3_) [17,18,19].

**Figure 3:**
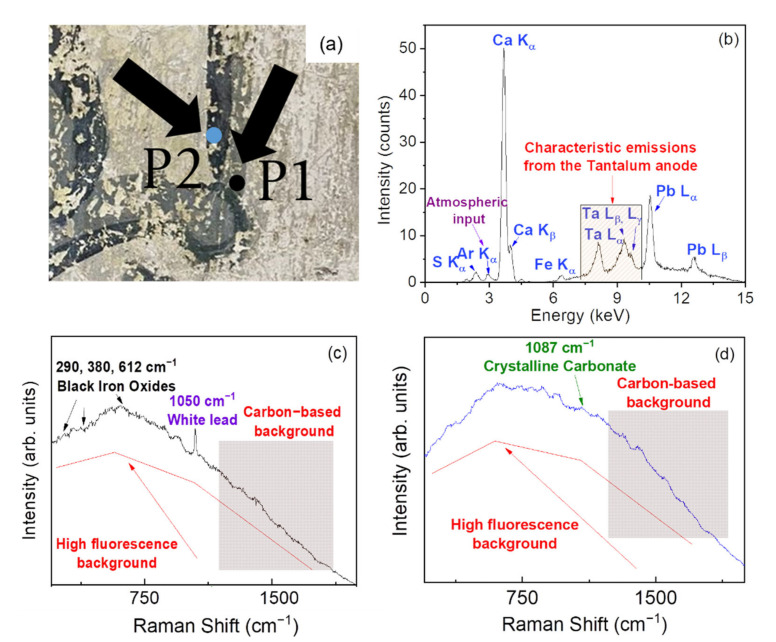
Photos of the points P1 (light gray) and P2 (dark gray) (**a**). XRF spectrum recorded on the point P1, reported as an example (**b**). Raman spectra recorded on points P1 and P2, respectively (**c**,**d**).

Going on, Figure 3 reports the XRF and Raman results concerning points P1 and P2 (Figure 3a), representative of a light and dark gray pigmented area of the Church, respectively.

A first inspection of Figure 3b reveals, for P1 point, the presence of Ca, Pb, Fe, and S in quite large amounts. Actually, XRF spectra collected on both P1 and P2 points can be overlapped, thus indicating a similar elemental composition. Moreover, in the case of point P1, Raman results (see Figure 3c) revealed overlapping with a high fluorescence background, a clear band at ~ 1050 cm^−1^, associated to white lead (2PbCO_3_·Pb(OH)_2_), and, in the 250–650 cm^−1^ range, different peaks compatible with the mentioned iron oxides [20].

From the results, the use of white lead as the main component to prepare the gray pigmented plaster [21] can be hypothesized, mixed with Fe_3_O_4_ and other iron oxides as black pigments. This can justify the high concentrations of Pb and Fe detected by XRF in the corresponding area. Moreover, as well known, white lead is unstable in the presence of sulfurous compounds and gases and reacts with them to form black lead (II) sulfide (PbS) [22]. This could also justify the presence of S in the elemental composition of P1.

Furthermore, the presence of a carbon-based compound was identified in the Raman spectrum of P1 through the observation of two strong contributions located at ~1300 and ~1600 cm^−1^. In particular, these signals are characteristic of sp2 carbon materials and have been traditionally assigned to the *D* (disorder) and *G* (graphitic) bands of amorphous carbon [23], respectively. Two different hypotheses could be made to explain the presence of these bands. The first hypothesis is that the carbon-based materials are connected to the presence of carbon nanoparticles due to the candle soot commonly used in liturgical ceremonies and, more in general, in church-related activities. A second hypothesis is that a carbon-based pigment was mixed with the black iron oxides in order to prepare the black pigment used as a base for the creation of the gray pigmented plaster.

The interpretation of the Raman spectrum of P2 (Figure 3d) is more complicated because of the presence of the high fluorescence background. A weak band at ~1087 cm^−1^ suggests the presence of a preparation ground made of calcium carbonate (CaCO_3_). However, the absence of the other characteristic peaks of calcite (the most common calcium carbonate) at ~162, ~282, ~712, and ~1435 cm^−1^ prevents us from defining with certainty the mineralogical nature of the carbonate [17,18,19]. In addition, even in this case, a carbonaceous/carbon-based material can be distinguished by its contributions at ~1300 and ~1600 cm^−1^ [23].

Based on the aforementioned considerations, a mixture of carbon-based pigment with a binder made of calcium carbonate (CaCO_3_) has been presumably employed to prepare the dark gray pigmented plaster. In addition, the light gray pigmented plaster can be described as a mixture of iron oxides, lead white, and a binder made, likely, of calcium carbonate (CaCO_3_).

**Figure 4:**
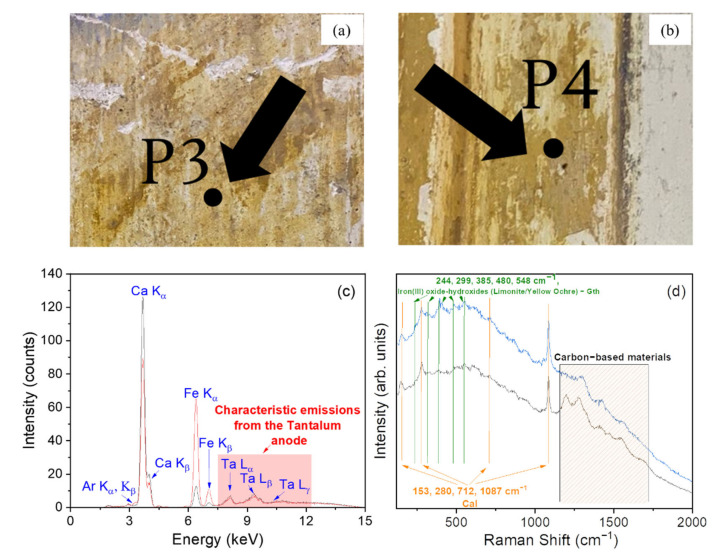
Photo of the point P3 (light yellow) (**a**). Photo of the point P4 (dark yellow) (**b**). XRF spectra recorded on the points P3 (black line) and P4 (red line) (**c**). Raman spectra recorded on the points P3 (gray line) and P4 (blue line) (**d**). In the figure, Gth: goethite; Cal: calcite.

Figure 4 reports the XRF and Raman results concerning points P3 (Figure 4a) and P4 (Figure 4b), representative of a light and dark yellow pigmented area of the Church, respectively.

The XRF spectrum collected on point P3 almost completely matched that of sample P4 (Figure 4c), indicating a similar elemental composition based on Ca and Fe as major components. Regarding Raman analysis (Figure 4d) performed on the corresponding areas, both spectra show the characteristic bands of calcite (at ~153, ~280, ~712, and ~1087 cm^−1^) [17,18,19], together with peaks in the 240–600 cm^−1^ range that can be assigned to some iron oxides and hydroxides [24,25,26,27], commonly included in the so-called yellow ochre pigment [27]. According to [28], ochres are natural colorant earths that have been widely used as artistic pigments since prehistoric times not only because of their natural abundance, normal easy extraction and preparation, or low commercial cost, but also because of their coloring capacity and stability under varied weather conditions, light, oxidation, and corrosion. Ochres contain variable quantities of iron oxides and hydroxides, among which hematite (αFe_2_O_3_) and goethite (αFeOOH) are the most frequent, and of white pigments (alumino-silicate as kaolinite or illite, quartz and calcium compounds as calcite, anhydrite, gypsum, or dolomite) [28,29]. In our case, the presence of goethite can be considered as responsible for the yellow coloration of the ochres.

In addition, the Raman measurements show, for both investigated points, the two strong bands located at ~1300 and ~1600 cm^−1^, interpreted as the characteristic pattern of carbonaceous and carbon-based materials as previously discussed. As explained before for the gray pigmented plasters, the bands related to the carbon-based materials can be ascribed to the presence of carbon nanoparticles due to the candle soot. Therefore, it can be concluded that the yellow pigmented plasters are the product of a mixture of yellow ochre with a binder made, presumably, of calcite.

**Figure 5:**
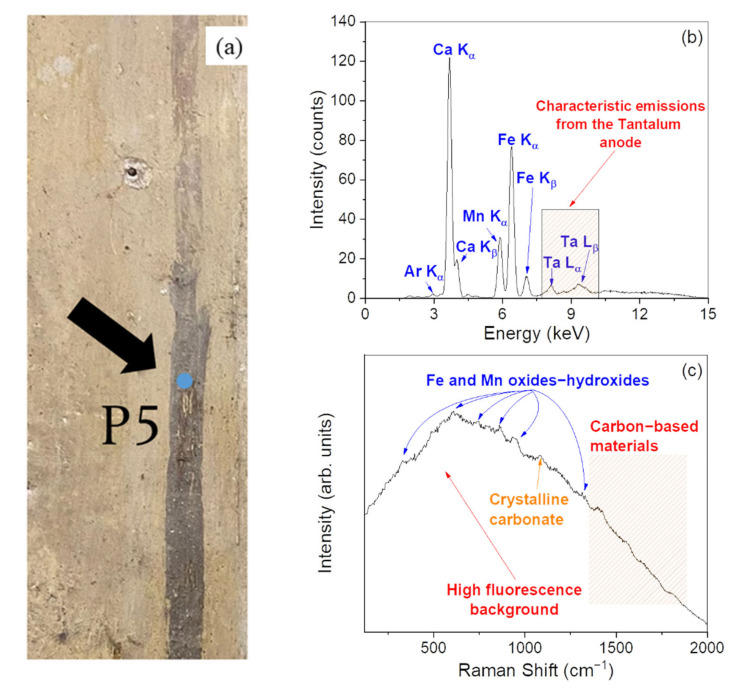
Photo of the point P5 (dark brown) (**a**). XRF spectrum recorded on the point P5 (**b**). Raman spectrum recorded on the point P5 (**c**).

Figure 5 reports the XRF and Raman results concerning the point P5 (Figure 5a), representative of a dark brown pigmented area of the Church.

In particular, XRF results (Figure 5b) indicate an elemental composition based on Ca, Fe, and Mn. In the corresponding Raman spectrum (Figure 5c), the weak peaks observed in the range from ~300 to ~1300 cm^−1^, can be ascribed to manganese and iron oxides–hydroxides [30,31], in agreement with the detection of Mn and Fe by XRF. These observations indicate that the investigated point was made by starting from the use of complex pigments derived from Mn-based and Fe-based products, such as the raw and/or burnt umber [32]. Finally, the observation in the Raman profile of a weak band at 1087 cm^−1^ indicates the presence of the preparation ground made likely of calcium carbonate (CaCO_3_). In addition, even in this case, a carbonaceous/carbon-based material can be distinguished by its contributions at ~1300 and ~1600 cm^−1^.

**Figure 6:**
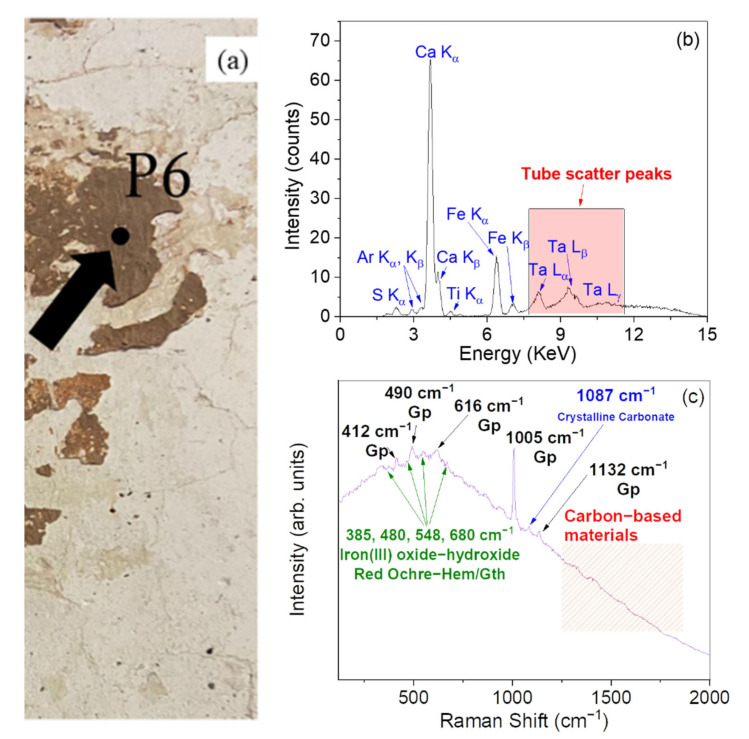
Photo of the point P6 (brown) (**a**). XRF spectrum recorded on the point P6 (**b**). Raman spectrum recorded on the point P6 (**c**). In the figure, Gp: gypsum; Hem: hematite; Gth: goethite.

Figure 6 reports the XRF and Raman results concerning the point P6 (Figure 6a), representative of a brown pigmented area of the Church.

On one hand, XRF results (Figure 6b) indicate a main elemental composition based on Ca, Fe, S, and Ti. On the other hand, Raman measurements (Figure 6c) revealed the characteristic bands of gypsum (~412, ~490, ~616, ~1005, and ~1132 cm^−1^) [33,34] together with a weak band at 1087 cm^−1^, ascribable to the presence of the preparation ground likely made of calcium carbonate (CaCO_3_). Moreover, peaks in the 350–700 cm^−1^ range can be assigned to some iron oxides and hydroxides, suggesting the application of red ochre [28,32]. Hematite is responsible for the red color of the ochres. The bands related to the carbon-based materials (at ~1300 and ~1600 cm^−1^) are weaker than the other samples analyzed.

According to the aforementioned considerations, the investigated point was reasonably realized by mixing red ochre with gypsum.

**Figure 7:**
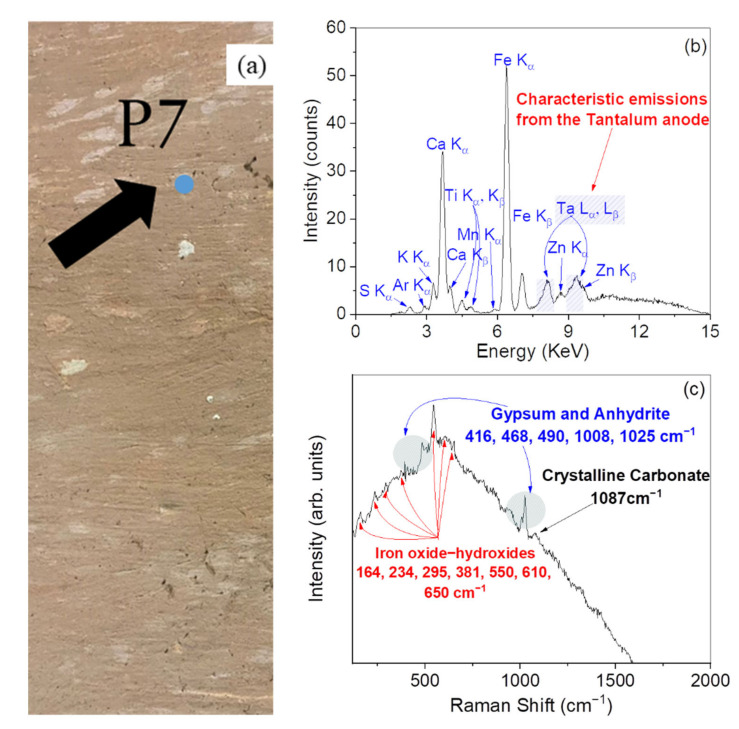
Photo of the point P7 (pink) (**a**). XRF spectrum recorded on the point P7 (**b**). Raman spectrum recorded on the point P7 (**c**).

Figure 7 reports the XRF and Raman results concerning the point P7 (Figure 7a), representative of a pink pigmented area of the Church.

The XRF spectrum (Figure 7b) shows the main peaks of Fe, Ca, K, Zn, Mn, and S. In particular, the content of K and Zn could be connected to the presence of clay minerals such as kaolinite or illite. The content of Zn could also be related to the employment of zinc oxide, often used as an additive in numerous materials [33] as, for instance, in pigments. The Raman measurements (Figure 7c) highlight the characteristic bands of gypsum and anhydrite (~412, ~490, ~616, ~1008, and ~1025 cm^−1^) [34,35]. Furthermore, the weak band at ~1087 cm^−1^ indicates the presence of the preparation ground likely made of calcium carbonate (CaCO_3_). Moreover, peaks in the 160–700 cm^−1^ range can be assigned to some iron and manganese oxides and hydroxides [24,25,26,27,28,29,30,31] commonly present in some red earths. The bands related to the carbon-based materials (at ~1300 and ~1600 cm^−1^) are very low compared to the other samples.

Based on the aforementioned considerations, the pink pigmented plaster can be considered as the product of a mixture of a red earth with a preparation ground made of gypsum, similarly to what has been observed for the point P6.

**Figure 8:**
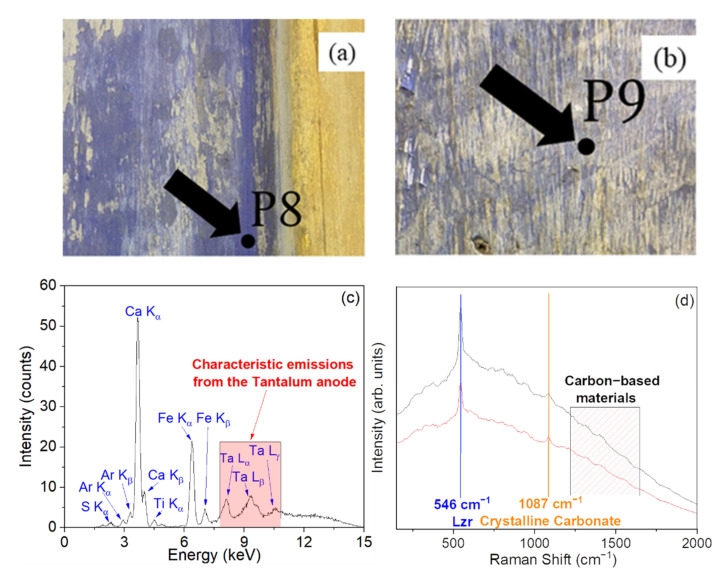
Photo of the point P8 (dark blue) (**a**). Photo of the point P9 (light blue) (**b**). XRF spectrum recorded on the point P8, reported as an example (**c**). Raman spectra recorded on the points P8 (grey line) and P9 (red line) (**d**). In the figure, Lzr: lazurite.

Figure 8 reports the XRF and Raman results concerning points P8 (Figure 8a) and P9 (Figure 8b), representative of a dark and light blue pigmented area of the Church, respectively.

In particular, XRF spectra of points P8 (Figure 8c) and P9 (not shown) can be overlapped, indicating a similar elemental composition, with the presence of Ca, Fe, Ti, and S in quite large amounts. Going on, Raman measurements in both points (Figure 8d) show two clear peaks at ~546 and ~1087 cm^−1^, which can be assigned to the presence of an ultramarine blue pigment [24,36] and to the calcium carbonate (CaCO_3_) from preparation ground, respectively.

There is much confusion in the literature between ultramarine blue (a pigment obtained from ground lapis lazuli), lapis lazuli (an aggregate of minerals), and lazurite (the most valuable ‘blue’ mineral component of the lapis lazuli) [37]. Lapis lazuli is a complex rock whose composition is defined by the presence of the mineral lazurite (Na,Ca)_8_(AlSiO_4_)_6_(SO_4_,S,Cl)_2_, which is responsible for its overall blue hue. Inclusions of several other minerals are also common, including pyrite (FeS_2_), calcite (CaCO_3_), diopside (CaMgSi_2_O_6_), forsterite (MgSiO_4_), and wollastonite (CaSiO_3_), in varying amounts [38]. Lazurite itself is a member of the sodalite group, which includes sodalite, nosean, and hauyne, and is typically considered a sulfur-rich hauyne [39]. The sodalite minerals contain frameworks of alternating silica and alumina tetrahedra creating large cubo-octahedral cages, known as β-cages; extra-framework cations (Ca^2+^, K^+^, or Na^+^), anions (Cl^−^, OH^−^, SO_4_^2−^, or Sn^−^), and neutral species (H_2_O) are entrapped within these cages [39].

Lazurite’s blue color is attributed to sulfur polyanion radicals trapped in the β-cage structure. Variations in color appear to be related to ratios between various sulfur species: the trisulfur radical (S_3_^−^) is mainly responsible for the blue color, but contributions from disulfur (S_2_^−^) and tetrasulfur (S_4_^−^) radicals can shift the color towards yellow or red, respectively [38].

Ultramarine is famous for having been the most expensive pigment. It was more expensive than gold during the Renaissance [40]. The pigment found its most extensive use at least since the 11th century in recipes for producing ‘blue’ colored inks employed in Muslim manuscripts, and in 14th and 15th century illuminated manuscripts and Italian panel paintings, often reserved for the cloaks of Christ and the Virgin [40].

Based on comparison with literature [24,36], it is possible to identify the ultramarine blue as synthetic. Interestingly, only in the year 1828, Jean-Baptiste Guimet, in France, and Friedrich August Kottig, in Germany, published the production process for a synthetic ultramarine [40]. In particular, it consisted of four steps: (i) clay activation, (ii) blending and heating of the raw materials, (iii) oxidation, (iv) purification and refinement. The raw materials used are kaolinitic clay, feldspar, anhydrous sodium carbonate, sulfur, and a reducing agent such as oil, pitch, or coal [35].

In this context, the ultramarine blue pigment found in the blue pigmented plaster in the investigated site played a key role in the understanding of the period of realization of the wall painting. In fact, as can be seen from Figure 8, no minerals commonly associated with the lapis lazuli were detected. Noteworthy, the presence of iron in the corresponding area, as detected by XRF, can be reasonably due to the raw minerals used for the production of the synthetic ultramarine blue. Fe-bearing minerals, as, for instance, illite, can be used in the industrial process to produce the pigment [41]. There is no trace of pyrite, the common Fe-bearing mineral associated with the natural lapis lazuli. The Church was built in 1757, and the synthetic ultramarine blue was commercialized only after the second decade of the XIX century.

All investigated pigmented plasters had an essential role in determining at least two different manufacturing phases. More in detail, pigmented plasters, with the exception of P6 and P7, belong to the first, and are characterized by a preparation ground made likely of calcium carbonate (CaCO_3_). In particular, for points P8 and P9 the finding of the synthetic ultramarine blue allowed us to hypothesize that such first phase belongs to an age later than the first two decades of 1800, which, as a matter of fact, could be considered as the original wall painting of the investigated Church. On the contrary, plasters with a preparation ground made of gypsum (P6 and P7) belong to a late phase of wall painting, which may be connected to the major restructuring interventions carried out during the XX century [42]. In addition, the aforementioned results allow us to restrict the time frame of historical seismic events which caused the fractures in the masonry structures of the Church [43].

Some crystalline deposit of salts, such as efflorescence, were also analyzed in the Church.

**Figure 9:**
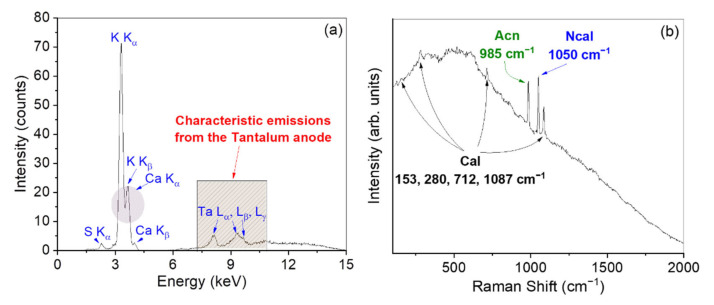
XRF spectrum recorded on the efflorescence (**a**). Raman spectrum recorded on the efflorescence (**b**). In the figure, Acn: arcanite; NCal: nitrocalcite; Cal: calcite.

On one hand, XRF results (Figure 9a) indicate the main composition of K, Ca, and S. On the other hand, Raman measurements (Figure 9b) reveal the characteristic peaks of calcite at ~153, ~280, ~712, and ~1087 cm^−1^ [17,18,19]; the peak at 1050 cm^−1^ is assigned to the mineralogical phase of nitrocalcite (Ca(NO_3_)_2_·4H_2_O) [43]; the peak at 985 cm^−1^ corresponds to the arcanite (K_2_SO_4_) mineralogical phase [44].

Calcium carbonate, or calcite, individuated in the efflorescence is the product of a secondary recrystallization. Indeed, calcium carbonate is the main component of lime mortars, and it can, however, dissolve and recrystallize under specific conditions. A common cause of degradation occurs when the carbon dioxide, from the atmosphere, slowly penetrates the mortars and converts the calcium hydroxide and cementing phases in calcium carbonate. This phenomenon is called carbonation and its effects are important in relation to the durability of mortars themselves [45].

One of the most commonly occurring alkali sulfates in mortars and cement is arcanite. During normal cement hydration reactions with water, these alkali sulfates can contribute to set control. However, hydration of alkali sulfates during cement storage can lead to lumping and flowability problems [45,46]. Alkali sulfate can affect the strength characteristics of cement and mortars. In addition, mortars and cement attacked by sulfates can also form arcanite [44,45,46]. This phase, as well as other sulfates, affects their durability. Sulfates can be considered as the predominant anions in efflorescence. Since these salts have a low hygroscopicity, they can quickly crystallize even in a humid climate and thereby cause the main deteriorations in mortars and cement [46]. Nitrates, such as nitrocalcite, are very soluble salts and can be related to micro-organisms and capillary water [46].

The information obtained from the analysis of the alteration products is sufficient to identify the development of alteration pathologies that afflict the entire building. Among these pathologies, the rising dump is the most recognized.

### 3.2. Mortars

**Figure 10:**
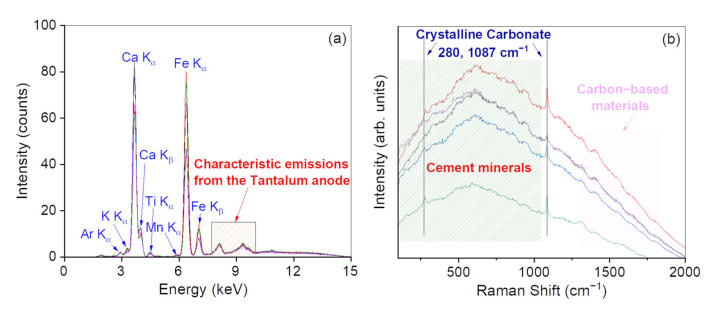
XRF spectra recorded on the mortars (**a**). Raman spectra recorded on the mortars (**b**). In the figure, M1: black line; M2: red line; M3: blue line; M4: pink line; M5: green line.

As far as mortars are concerned, XRF results (Figure 10a) indicate an elemental composition based on Ca, Fe, and traces of Ti, K, and Mn.

Going on, all Raman spectra (Figure 10b) show clear peaks at ~280 and ~1087 cm^−1^, suggesting the presence of calcium carbonate (CaCO_3_) [17,18,19]. Even in this case, carbonaceous/carbon-based materials can be distinguished by the observation of two strong bands located at ~1300 and ~1600 cm^−1^. Interestingly, all the contributions falling in the 150–1000 cm^−1^ range can be assigned to the presence of cement and clay minerals. In this range there is significant overlap between the mineral phases with a large fluorescence effect as suggested by [47]. Silicates provide a spectrum which is dominated by the internal modes of SiO_4_^4-^ tetrahedral units. In our case, it is possible to distinguish the symmetric (ν_2_) and antisymmetric (ν_4_) bending of the O-Si-O bonds, centered at ~448 and ~607 cm^−1^, respectively, together with the symmetric (ν_1_) and antisymmetric (ν_3_) stretching of Si–O bonds at ~777 and ~935 cm^−1^ [48]. These results support the hypothesis that the investigated mortars are composed of lime and clay materials; the reaction between these two components leads to the formation of low-soluble calcium silicate hydrates or calcium aluminate hydrates, which are responsible for the hydraulic behavior of the mortar. The cement hydration process can be represented by a set of chemical equations that describes the hydration of the main cement mineral phases C_3_S, C_2_S, C_3_A, and C_4_AF (the nomenclature used in this article for cement is C = CaO, S = SiO_2_, H = OH, A = Al_2_O_3_, F = Fe_2_O_3_). The bands in the 200–400 cm^−1^ region probably belong to Ca–O vibrations. Furthermore, the bands in the region between 525 and 550 cm^−1^ can be assigned to O–Si–O bending modes of C_3_S/C_2_S. A broad feature near 730 cm^−1^ is composed of vibration of C_4_AF at 737 cm^−1^ and of C_3_A at 754 cm^−1^. The stretching vibration bands of Si–O characteristics of C_3_S and β-C_2_S phases can be seen in the Raman spectrum in the 800–900 cm^−1^ region. Finally, the band at ~854 cm^−1^ can be assigned to C_2_S/C_3_S [49].

From the geological setting surrounding the city of Tortorici, it is known that the lithology of different formations is mainly composed of phyllosilicates (mica and clay minerals), quartz, albite, calcite, and minor amounts of K-feldspar [50], displaying for the mineralogical composition of clay minerals a mixture of illite-rich assemblages, chlorite, kaolinite, and subordinate amounts of mixed-layered clay minerals [50].

According to [51,52], in old buildings and archaeological sites, also geographically connected to the city of Tortorici [52], the use of hydrated lime mortars with several additions of, for instance, heat treated clays and ashes was a common choice, thus providing mortars with hydraulic properties and improving the mechanical behavior of mortar itself through the occurrence of pozzolanic reactions.

As final remark, it is worth highlighting that the methodology employed can be helpful in view of possible subsequent investigations and modeling stages. Moreover, the obtained results furnished preliminary information useful in view of the vulnerability assessment of the building.

## 4. Conclusions

In this paper, plasters and mortars of the *Church of the Annunciation* (Tortorici, Sicily) were characterized by means of portable XRF and Raman instrumentations.

In particular, concerning the pigmented plasters, two different typologies were distinguished based on the presence of calcium carbonate and gypsum, respectively. According to this classification, two different manufacturing phases were revealed. The first, belonging to an age later than the first two decades of 1800, was considered as the original wall painting of the investigated Church, while the second to a late phase, which may be connected to the major restructuring interventions carried out during the XX century.

Going on, information obtained from the analysis of joint mortars revealed a general homogeneity in composition. The identification of minerals connected to calcium silicate hydrates or calcium aluminate hydrates suggests the use of heated local raw materials in the preparation of the investigated mortars.

Nonetheless, further study of, and research into, this site is needed, and it will be developed in the future. In particular, (i) infrared thermography is planned to be used to detect subsurface features, such as defects and anomalies, based on temperature differences and to map moisture distribution and detect areas of moisture-related deterioration in structures. (ii) A more detailed sampling campaign of mortars needs to be planned, also by micro-coring of masonry walls to identify the state of health of the mortars themselves and their related potential degradation products. (iii) Finally, 2D and 3D digital models of the monument using digital photogrammetry need to be created in order to map the different restoration phases and create tools for the usability of the studied site.

## Figures and Tables

**Table 1:** A list of the analyzed points, together with a brief macroscopic description.

ID	Description
PG	White colored preparatory ground
P1	Pigmented plaster—light gray
P2	Pigmented plaster—dark gray
P3	Pigmented plaster—light yellow
P4	Pigmented plaster—dark yellow
P5	Pigmented plaster—dark brown
P6	Pigmented plaster—brown
P7	Pigmented plaster—pink
P8	Pigmented plaster—dark blue
P9	Pigmented plaster—light blue
ALT	Crystalline deposit of salts (efflorescence)
M1	Gray mortar joint
M2	Gray mortar joint
M3	Gray mortar joint
M4	Gray mortar joint
M5	Gray mortar joint

## Data Availability

The data presented in this study are available on request from the corresponding author.
